# Finger-specific effects of age on tapping speed and motor fatigability

**DOI:** 10.3389/fnhum.2024.1427336

**Published:** 2024-09-25

**Authors:** Caroline Heimhofer, Amira Neumann, Ingrid Odermatt, Marc Bächinger, Nicole Wenderoth

**Affiliations:** ^1^Neural Control of Movement Lab, Department of Health Sciences and Technology, ETH Zurich, Zurich, Switzerland; ^2^Center for Neuroscience Zurich (ZNZ), Federal Institute of Technology Zurich, University and Balgrist Hospital Zurich, University of Zurich, Zurich, Switzerland; ^3^Future Health Technologies, Singapore-ETH Centre, Campus for Research Excellence and Technological Enterprise (CREATE), Zurich, Switzerland

**Keywords:** Fatigue, behavior, in-field experiment, aging, motor fatigability, tapping speed, motor slowing

## Abstract

**Introduction:**

Increased motor fatigability is a symptom of many neuromuscular and neurodegenerative disorders. However, it is difficult to pinpoint pathological motor fatigability, since the phenomena has not yet been fully characterized in the healthy population. In this study, we investigate how motor fatigability differs across age. Given that many disorders involve supraspinal components, we characterize motor fatigability with a paradigm that has previously been associated with supraspinal mechanisms. Finger tapping at maximal speed results in a rapid decrease in movement speed, which is a measure of motor fatigability.

**Methods:**

We collected finger tapping data in a field experiment from the general population with a smartphone app, and we investigated age differences in maximal tapping speed, as well as the decrease in tapping speed for the index, middle, and little fingers.

**Results:**

We found that the maximal tapping speed differed significantly between young (18–30 years, *n* = 194) and aged (50–70 years, *n *= 176), whereas the fatigability-induced relative decrease in movement speed did not differ between the age groups (average decrease: 17.0% ± 6.9% (young) vs. 16.5% ± 7.5% (aged) decrease). Furthermore, tapping speed and motor fatigability depended on which finger was used.

**Discussion:**

These findings might relate to dexterity, with more dexterous movements being more resistant to fatigue. In this study, we provide a characterization of motor fatigability in the general population which can be used as a comparison for clinical populations in the future.

## Introduction

Motor fatigability poses a challenge in many neuromuscular and neurodegenerative disorders (Friedman et al., 2016; Manjaly et al., 2019). Behaviourally, it can manifest as a decline in motor performance, which may make it difficult to maintain activities of daily living for extended periods. Motor fatigability might be pathologically elevated in certain patient groups causing their quality of life to diminish, for example, due to restricted mobility. The prevalence of neuromuscular and neurodegenerative disorders increases with age (Wyss-Coray, 2016). However, distinguishing physiological from pathological levels of fatigability is not trivial. As a reference to characterize clinical populations, a normative data set for well-defined motor tasks that can be tested in a scalable manner and outside of clinical settings may be helpful. However, these normative data for motor fatigability are not yet available and thus in-depth characterization of the effects of ageing in a large population sample is still required (Correia et al., 2022).

Previous research has revealed inconsistent effects of ageing on motor fatigability. Different studies have reported decreased, increased, or similar levels of motor fatigability in young versus aged participants, depending on factors such as contraction mode, the involved muscle group, or the applied fatigue index (see reviews: Christie et al., 2011; Krüger et al., 2018; Paris et al., 2022). For example, young participants show more fatigability compared to aged participants during isometric contraction protocols (Christie et al., 2011), whereas during unconstrained dynamic contractions, which is argued to be a more natural movement, young participants fatigue less (Paris et al., 2022). These findings suggest that age differences in motor fatigability could be task-specific (Bigland Ritchie et al., 1978; Enoka and Stuart, 1992; Hunter et al., 2016). However, it is also possible that ageing differently affects various neurobiological substrates that contribute to fatigability. It is well known that motor fatigability is not uniquely induced by the peripheral neuromuscular system and that central mechanisms are critical, for example, through suboptimal cortical drive (e.g., Bächinger et al., 2019; Gandevia, 2001; Gandevia et al., 1996; Post et al., 2009; Smith et al., 2007; Søgaard et al., 2006; Taylor et al., 2006; Teo et al., 2012). Also, the relative contribution of supraspinal versus spinal and neuromuscular mechanisms to fatigability has been shown to differ depending on task demands (Arias et al., 2015; Madrid et al., 2016, 2018; Rodrigues et al., 2009). While it is well known that the peripheral neuromuscular system generally changes with age (Hunter, 2018; Hunter et al., 2016), age-specific changes of supraspinal fatigability mechanisms and their effects on motor performance are less well investigated.

A paradigm which has been shown to be predominantly associated with supraspinal fatigability mechanisms, is the execution of low-force fast repetitive movements, for example during fast finger tapping. The marginal peripheral involvement in fast finger tapping is evidenced by a studies showing that muscle contractile properties show no signs of impairments pre and post a fast finger tapping task (Madinabeitia-Mancebo et al., 2021) and that maximal voluntary muscle contractions show no significant changes in force after the task (Madrid et al., 2018; Rodrigues et al., 2009). Also, voluntary activation to the muscle and spinal excitability were not altered after fast finger tapping at maximal speed (Arias et al., 2015; Kluger et al., 2012; Madrid et al., 2016, 2018). Fast repetitive finger tapping has been frequently used for diagnosing and monitoring motor impairment, particularly, in Parkinson’s disease (Bartzokis et al., 2010; Haaxma et al., 2010; Jiménez-Barrios et al., 2023; Lou et al., 2003; Shimoyama, 1990; Taylor Tavares et al., 2005) and multiple sclerosis (Barrios et al., 2020; Bonzano et al., 2013; Gulde et al., 2021; Kane et al., 2007). Here we use this paradigm to quantify motor fatigability. We define motor fatigability as a decrease in movement speed, so-called motor slowing, which occurs when tapping at maximal speed must be maintained over a longer period. We proposed that motor slowing is at least partly caused by a gradual release of inhibition (including a breakdown of surround inhibition) in the sensorimotor cortex which is associated with elevated muscular co-activation (Bächinger et al., 2019). Neurodegenerative disorders, such as Parkinson’s disease or multiple sclerosis, have also been associated with disturbed surround inhibition in the motor system (Belvisi et al., 2021; Shin et al., 2007). Therefore, motor slowing lends itself well as a paradigm to investigate supraspinal aspects of motor fatigability via simple movements such as finger tapping, which can be performed outside of the lab.

In our study, we showed that it is feasible to quantify motor fatigability outside of the laboratory via a mobile digital approach using a smartphone or tablet and acquired a first dataset in 194 young healthy participants (18–30 years) and 176 aged healthy participants (50–70 years). We addressed the question of how aging affects motor fatigability using a prolonged tapping task at maximal speed that has been shown to evoke fatigability via supraspinal rather than spinal or neuromuscular mechanisms. We investigated if there is a difference in maximal tapping speed and motor slowing between healthy young and aged participants. The fast-tapping task was performed with different fingers to investigate a potential interaction with dexterity.

## Materials and methods

### Participants

Data of 450 participants (18–30 years and 50–70 years, 233 female, 36 left-handed) was collected in a field experiment using a mobile device. After the data quality check (see Data Processing), 370 participants (196 females, 29 left-handed) were included in the final analysis. All participants reported being free from neurological, psychiatric, or musculoskeletal disorders, not suffering from arthritis or limited mobility in the fingers of the dominant hand, and not taking any psychopharmacological medication. This study was approved by the ETH Zurich Ethics Commission (2020-N-185). All participants gave informed consent in accordance with the declaration of Helsinki before participating in the study.

### Experiment setup and procedure

The participants performed the experiment outside of the lab under supervision. The experiment was performed via a custom-made app programmed in Unity 2020.1.13f1 (Unity Technologies), which was administered through a smartphone or tablet.

**Figure 1:**
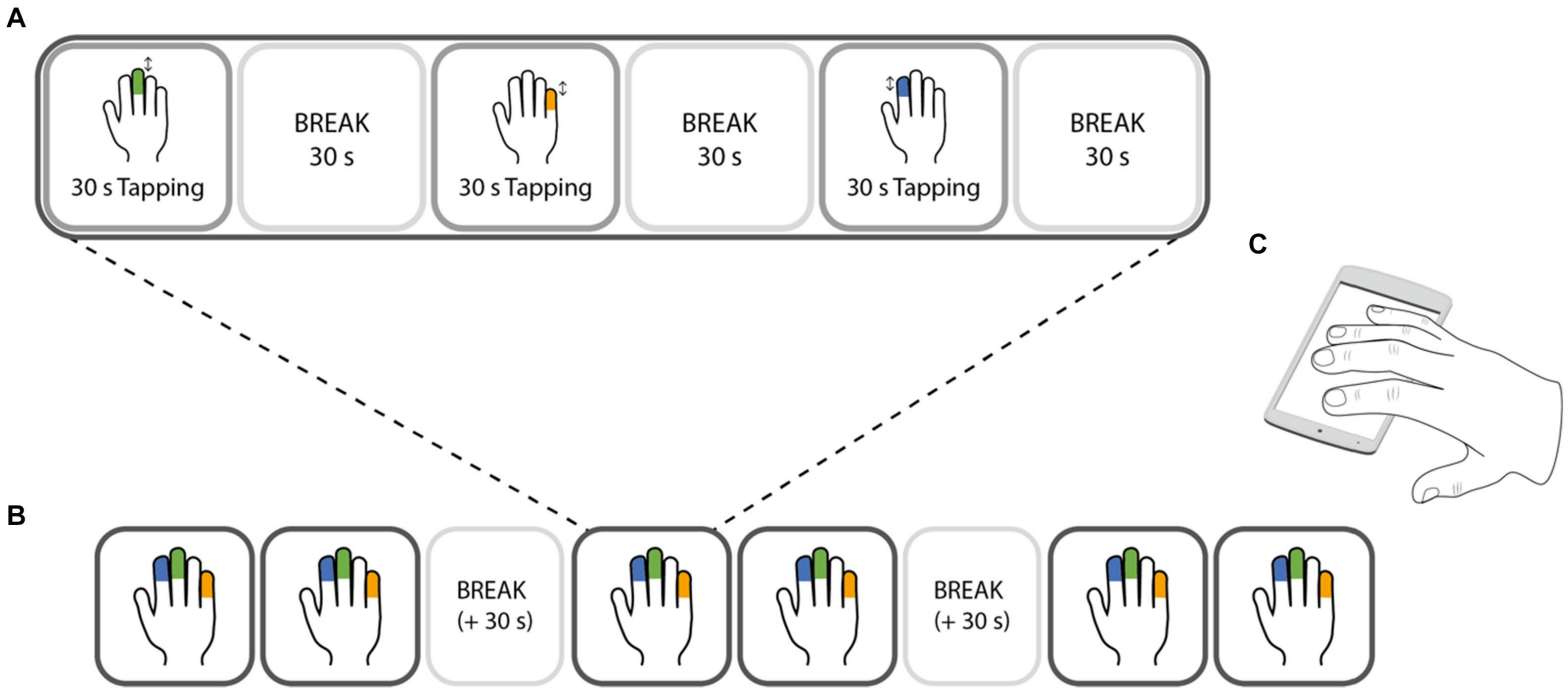
Design of the experimental task. Finger tapping was performed with either the index (blue), middle (green), or little (orange) finger. Only one finger was tapping at a time, while the other fingers (except the thumb) were resting on the screen of the testing device **(C)**. The experiment consisted of 6 blocks **(B)**, in which each finger had one trial. Each trial **(A)** consisted of 30 s of tapping at maximal speed with one finger, followed by 30 s break. During the break periods, all 4 fingers were resting on the screen. The order of the fingers was randomized within a block, i.e., pseudorandomised across the whole task.

The experiment consisted of 18 trials in total, in which participants tapped as fast as possible with either the index, middle, or little finger of the dominant hand (six trials per finger). The trials were split into six blocks, in which every finger had to tap for one trial. Within the blocks, the order of the fingers was randomized. Each trial consisted of 30 s tapping followed by a 30 s break. After block 2 and block 4 were completed, the break was extended by 30 s (Figure 1).

During the experiment, participants saw a progress bar on the screen which displayed the remaining and completed trials, and a timer bar, that indicated the remaining time of the trial or break. After first finger placement on the screen, visual circles guided the participants to keep the finger position stable. Shortly before and during the trial, the finger to tap with (index, middle, or little) was indicated. To additionally motivate the participants, a tap counter displayed the number of taps that the participant performed during a trial.

Participants were instructed (through the field experimenter and the app) to perform the task while sitting on a chair with the device resting on a table, in an environment that allowed them to focus on the task. They were further instructed to (i) only tap with the instructed finger as fast as possible from the start of the tapping period until the end while (ii) keeping the other fingers on the screen (except for the thumb). The non-tapping fingers were instructed to be kept on the screen. This allowed us to check that the participants were tapping with the instructed finger. Further, they were also instructed to not change their tapping strategy during the tapping period: The wrist position had to be kept stable and the device was not allowed to be shifted around on the table. During the break period, the participants were instructed to rest their fingers on the screen. This allowed us to detect unwanted finger movements during the break.

Before starting the actual task, participants familiarized themselves with the task by tapping with each finger for 15 s, followed by a 15 s break. The participants could repeat this familiarization task as often as they considered necessary.

### Data preprocessing

The data was preprocessed in MATLAB R2020b (MathWorks). The raw data from the smartphone or tablet consisted of touches on the screen for each time frame at a 50 Hz frequency. From the x and y components of each touch, we were able define which finger belonged to which touch. This then allowed tracking the tapping behavior of each finger. To assure that the participants had performed the task as instructed, we included several quality checks. The following criteria were checked: (1) Perform task at rest: As participants were instructed to do the task at rest while sitting on a chair, accelerometer data of the testing device was checked. A trial was excluded if too much motion of the device was detected after smoothing with a moving average filter of 0.25 s (i.e., accelerometer values with *x* > 0.03, *y* > 0.045, and *z* > 0.4). (2) Focus on experiment: To ensure that participants were focused on the trial start, we excluded trials in which participants took more than 1.5 s to start tapping after the starting cue was given. (3) Tap with instructed finger and keep other fingers on the screen: During the tapping period, participants had to (i) tap with the correct finger, and (ii) have 3 or 4 fingers on the screen (less than 3 fingers were allowed for maximally 3 s). The fingers were identified based on the x-coordinates from the device touchscreen. (4) Tap as fast as possible from the beginning: As participants were also instructed to tap as fast as they can from the beginning on, trials were excluded if the maximal tapping frequency was not attained within the first 10 s. (5) Do not change tapping strategy: Changing ‘tapping strategy’ throughout the trial (e.g., additionally engaging the wrist in tapping) may result in speeding up toward the end of the trial. We thus removed trials in which the minimal tapping frequency was not attained within the last 10 s, to make sure the tapping strategy was maintained. (6) Do not tap during rest period: During the rest periods, we checked that the participants did not continue tapping and that they always kept their fingers on the display. We allowed for a period of 3.5 s with less than 4 fingers on the screen starting 5 s into the rest period. (7) Technical failures: To capture technical failures of the app or the testing device, we removed individual inter-tap intervals from trials, if there was missing data for more than 2 s (i.e., the inter-tap interval was larger than 2 s). A maximum of 3 × 2 s or 5 s in a row of missing data was considered acceptable. If more data was missing, the trial was excluded from further analysis. The percentage of trials excluded for each criterion can be found in Table 1. If more than 3 trials per finger were removed, the whole finger was excluded from the analysis and if all three fingers were excluded from the analysis, the participant was not further analyzed. 38 young (18–30 years) participants (16.4%) and 42 aged (50–70 years) participants (19.3%) were completely excluded.

**Table 1:** Criteria for quality check to ensure participants had performed the task as instructed.

Criteria	Excluded trials (% of 8,100 in total)
1.Perform task at rest	3.6
2.Focus on experiment	< 0.1
3.Tap with instructed finger and keep other fingers on the screen	1.8
4.Tap as fast as possible from the beginning	13.3
5.To not change tapping strategy	17.6
6.Do not tap during rest period	12.0
7.Technical failure	< 0.1

**Figure 2:**
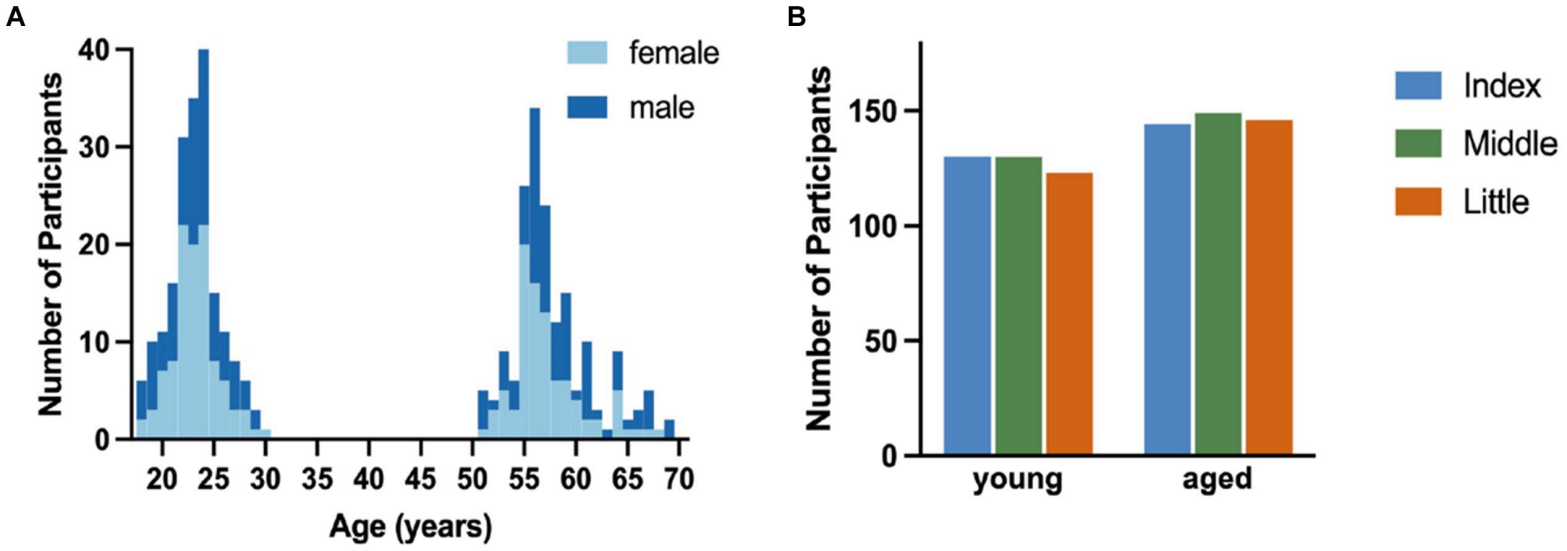
Overview of included participants. **(A)**. Distribution of participants across age range for male (dark blue) and female (light blue) participants. **(B)**. Count of participants in each age group for each finger with the index finger in blue, middle finger in green, and little finger in orange.

After data preprocessing, 274 participants had valid index finger trials, 279 participants had valid middle finger trials, and 269 had valid little finger trials (Figure 2B). For statistical analysis, the tapping frequencies of each trial were binned into 6 × 5 s time bins and averaged across valid trials for each finger.

Participants were split into two age groups: young (18–30 years, 194 participants, 106 female, 15 left-handed) and aged (50–70 years, 176 participants, 90 female, 14 left-handed). The age and sex distributions as well as the number of valid participant samples for the individual fingers are visualized in Figure 2.

### Statistical analysis

Statistical analysis was done in R Studio (Version 4.3.2). Firstly, to investigate whether we see a difference in maximal tapping speed between the age groups and tested fingers, we set up a linear mixed-effects model with *Maximal Tapping Speed* as dependent variable and *Age Group* (young, aged) and *Finger* (index, middle, little finger), and their interaction as independent variables. As tapping speed has been shown to differ between males and females (e.g., Hubel et al., 2013a,b; Shimoyama, 1990), we added *Sex* and *Sex x Finger* interaction as covariate to the model. Further, a random intercept of *Participant* was used.

To then find whether motor slowing differs across the age groups and tested fingers, we calculated the relative decrease in tapping speed from maximal to minimal (in percentage) tapping speed for each finger per participant:


$$\text{Relative}\;\text{Decrease}\;\left(\%\right)=\frac{\text{Tapping}\;\text{Spee}d_{\text{min}}\times 100}{\text{Tapping}\;\text{Spee}d_{\text{max}}\;}$$

We used another linear mixed-effects model on the dependent variable *Relative Decrease* as a function of the factors *Age Group* (young, aged) and *Finger* (index, middle, little finger), their interaction, the covariate *Sex*, *Sex x Finger* interaction, and the random intercept of *Participant*. The same analysis was performed on the *Absolute Decrease* in tapping speed, which was calculated from maximal to minimal tapping speed (not in percentage).

Finally, we aimed to investigate in depth how the course of motor slowing (i.e., the tapping speed in dependence of time) changes across the age groups and tested finger. For that, we fitted a model that consisted of the dependent variable *Tapping Frequency* as a function of the within-factors *Time* (6 × 5 s bins), and *Finger* (index, middle, little finger), the between factor *Age Group* (young, aged), the covariate *Sex*, the *Sex x Finger* interaction, and a random intercept of *Participant*.

In all models, outliers were detected and removed using the robustbase package (Maechler et al., 2023). The lme4 package (Bates et al., 2023) was used for the linear mixed effects model and type III ANOVA tables were calculated with the lmerTest package (Kuznetsova et al., 2020). Effect sizes were calculated as partial eta squared ($\eta_{p}^{2}$) for the significant factors using the effectsize package (Ben-Shachar et al., 2023). Post-hoc comparisons were performed using the multcomp package, which corrected for family-wise error rate (Hothorn et al., 2023).

## Results

### Maximal tapping speed changes across age and fingers

**Figure 3:**
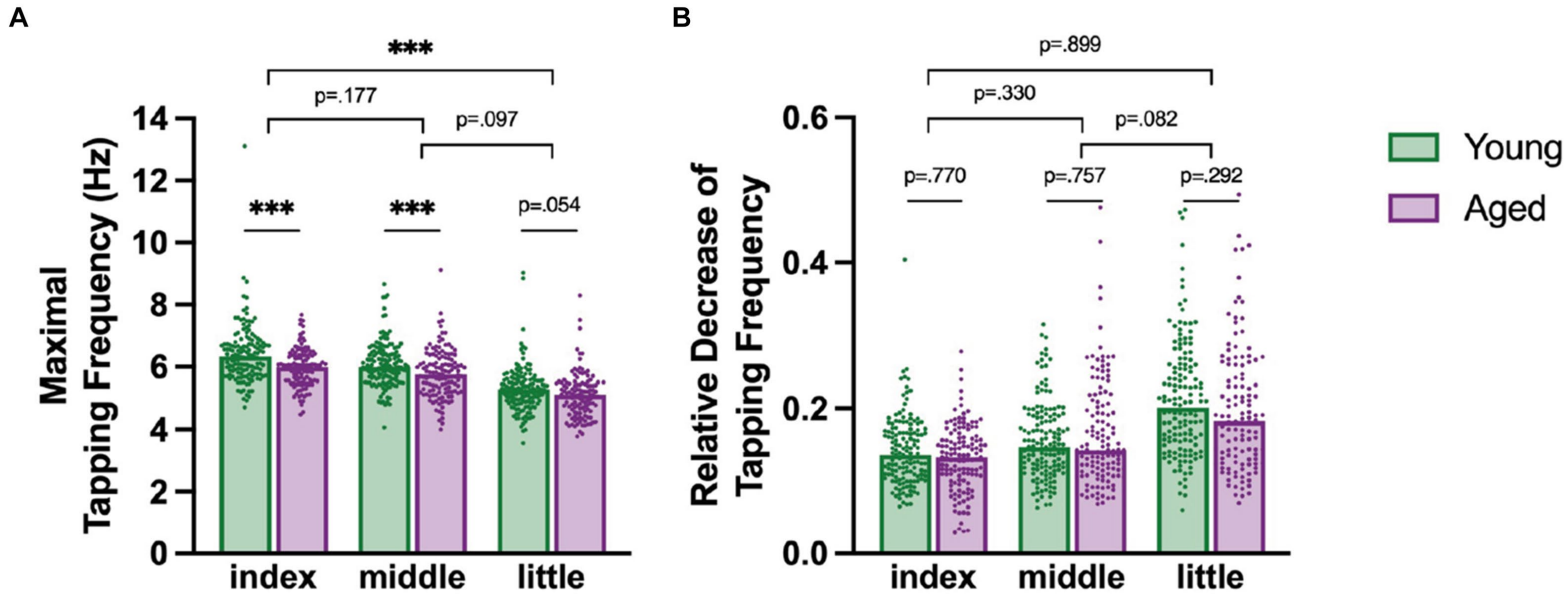
**(A)** Maximal tapping speed for fingers and age groups with young in green and aged in purple. **(B)** Motor slowing as a relative decrease of tapping speed from maximal to minimal tapping speed for different fingers for each age group. Each dot represents a participant in the specific category. Significant post-hoc comparisons are indicated with asterisk: *** *p* < 0.001. The short lines indicate comparisons between the age groups, whereas the longer lines indicate comparisons of the difference between the age groups across the fingers.

We investigated whether maximal tapping speed differed between fingers and age groups (Figure 3A). Tapping with the index finger was fastest and tapping with the little finger slowest, but this effect further differed between age groups [*Age Group x Finger* interaction, *F*_(2,465.24)_ = 9.115, *p* < 0.001, $\eta_{p}^{2}$ = 0.04] with young participants tapping significantly faster than aged participants with the index and the middle fingers (post-hoc *z* ≥ 4.186, *p* < 0.001), but not with the little finger, in which only a trend (*p* < 0.1) was detectable (*post hoc*, z = 2.529, *p* = 0.054). Further, analysis of the covariate *Sex* revealed that males tapped faster at their maximal speed than females [*F*_(1,350.50)_ = 9.614, *p* < 0.05, $\eta_{p}^{2}$ = 0.03].

### Relative decrease in tapping speed changes across fingers and age groups

Next, we tested whether the relative decrease in tapping frequency, a simple index of motor slowing, differs between fingers and between young (18–30 years) and aged participants (50–70 years, Figure 3B).

There was a general difference between fingers which varied between the age groups, as indicated by a significant *Age Group x Finger* interaction [*F*_(2, 485.67)_ = 3.152, *p* < 0.05, $\eta_{p}^{2}$ = 0.01] and a significant main effect of *Finger* [*F*_(2, 490.96)_ = 100.598, *p* < 0.001, $\eta_{p}^{2}$ = 0.32]. Overall, the index finger had the smallest reduction in tapping speed, followed by the middle and then the little finger. Post-hoc comparisons however revealed that the age differences and finger pairs show no significant differences in relative decrease (post-hoc *|z|* ≤ 2.368, *p* ≤ 0.082). Further, also male and female participants slowed similarly and there was no significant effect of *Sex* [*F*_(1, 310.41)_ = 2.274, *p* = 0.133].

The same analysis was repeated using the absolute decrease in tapping speed (instead of the relative decrease) as the dependent variable. These results revealed a significant main effect of *Age Group* [*F*_(1, 327.03)_ = 3.9243, *p* < 0.05, $\eta_{p}^{2}$ = 0.01] and a significant main effect of *Finger* [*F*_(2, 488.99)_ = 32.216, *p* < 0.001, $\eta_{p}^{2}$ = 0.12]. Post-hoc tests here revealed a significant difference between all finger pairs [*post-hoc z* ≥ 3.433, *p* ≤ 0.01]. The figure can be found in the Supplementary Figure S1.

### Motor slowing curve change across fingers and age groups

**Figure 4:**
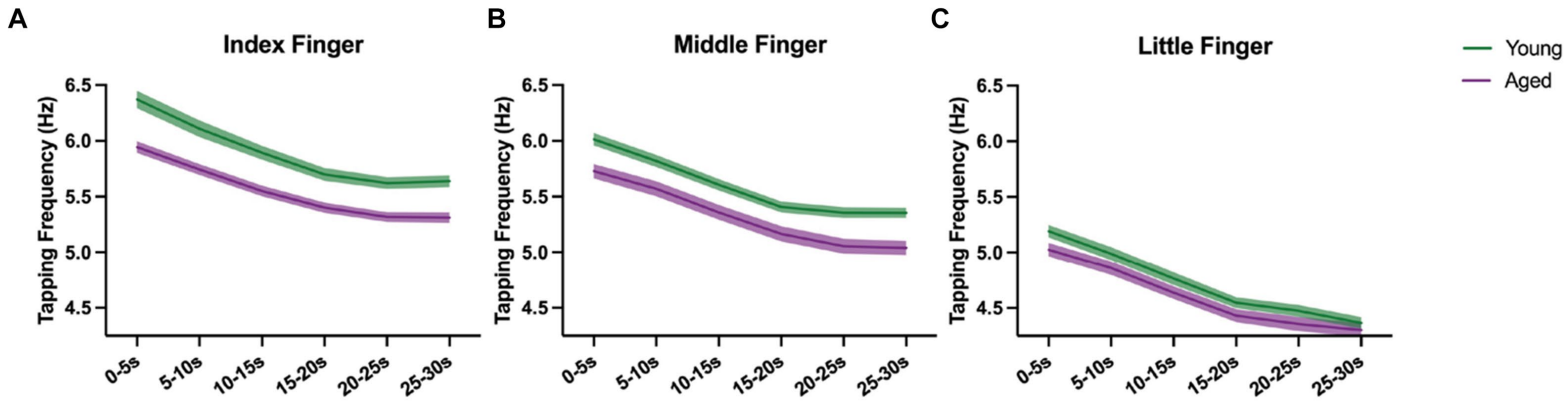
Course of motor slowing for aged (purple) and young (green) with mean and standard error, shown for index **(A)**, middle **(B)**, and little finger **(C)** separately.

We assessed whether the time course of the decrease in tapping frequency differs between age groups and tested fingers (Figure 4).

Tapping frequency decreased consistently over time in both age groups and for all fingers. Besides significant main effects of *Time*, *Finger*, and *Age Group* (all *p* < 0.001), we also found significant *Time x Finger* [*F*_(10, 4510.9)_ = 2.099, *p* < 0.05, $\eta_{p}^{2}$ = 0.04] and *Age Group x Finger* [*F*_(2, 4573.4)_ = 80.533, *p* < 0.001, $\eta_{p}^{2}$ = 0.03] interaction effects. As revealed in the analysis of the relative decrease, the *Time x Finger* interaction can be interpreted that the decrease is dependent on the finger, but the shape of the curves is similar for young and aged participants (no significant *Time x Finger x Age Group* [*F*_(10, 4510.9)_ = 0.581, *p* < 0.83] interaction). Also, the *Age Group x Finger* interaction may reflect the results from the maximal tapping speed analysis which showed that the maximal tapping speed between young vs. aged changed across fingers. Similarly, the average tapping speed differs between young vs. aged and changes across fingers.

We also found a significant main effect of the covariate *Sex* [*F*_(1, 361.7)_ = 10.030, *p* < 0.01, $\eta_{p}^{2}$ = 0.03] and the *Sex x Finger* interaction [*F*_(2, 4578.3)_ = 11.129, *p* < 0.001, $\eta_{p}^{2}$ = 0.004], indicating that males tap faster than females on average and that the extent differs depending on the finger (Supplementary Figure S2).

## Discussion

We investigated how aging affects supraspinal mechanisms of motor fatigability by exploring if there is a difference in motor slowing between healthy young and healthy aged participants. Younger participants tapped significantly faster than aged participants but, interestingly, we found no difference in motor slowing in terms of relative decrease in tapping speed between young and aged participants. Also, we found no significant difference in the time course of the motor slowing curves between the age groups, but instead found that the maximal tapping speed and the decrease in tapping speed were dependent on the finger that was tapping.

We found that the young tapped consistently faster than aged, particularly with the index finger. This is supported by a large body of literature that reports higher tapping speeds for young compared to aged participants (Aoki et al., 2019; Aoki and Fukuoka, 2010; Arias et al., 2012; Ashendorf et al., 2009; Bartzokis et al., 2010; Bornstein, 1985; Godefroy et al., 2010; Hubel et al., 2013a; Kwon et al., 2022; Shimoyama, 1990). Interestingly, we found that age differences in tapping speed depended on which finger was used. We found a clear age effect for the index and middle finger, which is in line with two studies by Aoki et al. (2019) and Aoki and Fukuoka (2010). While Aoki et al. (2019) found consistent differences in tapping speed between old and young in all digits (Aoki and Fukuoka, 2010), we only found a trend toward an age difference in the little finger. This discrepancy may stem from the ages of the participants in the older age group. Compared to our study, in which participants in the aged group were between 50 and 70 years of age, Aoki et al. tested older participants with an age range of 65–77 years (Aoki et al., 2019; Aoki and Fukuoka, 2010). Additionally, our study with 194 participants in the young and 174 participants in the aged group had a much larger sample size. Thus, it is possible that the findings of Aoki et al. who tested *N* = 14 (Aoki and Fukuoka, 2010) and *N* = 10 (Aoki et al., 2019) participants per age group did only partly generalize to a larger sample.

In our study, the decrease in tapping speed, the maximal tapping speed, and the time course of motor slowing depended on which finger executed the tapping task. The index finger had the highest maximal tapping speed and the smallest decrease, whereas the little finger had the lowest maximal tapping speed and the largest decrease, with the middle finger results being in between. These differences may arise due to functional differences in finger usage. The index finger, which is the most independently used finger of the ones we tested (Ingram et al., 2008), is mostly used for precision tasks (Bain et al., 2015). The little finger is recruited for power grip (Bain et al., 2015) and is often used in combination with ring and middle fingers (Ingram et al., 2008). This natural coupling might therefore limit tapping speed with the little finger while keeping the other fingers on the tablet. Furthermore, it has been shown that mental fatigue reduces overall dexterity (Duncan et al., 2015; Valenza et al., 2020). Broken down to individual fingers, fingers with more dexterity (index and middle) may hence be more robust to motor fatigability. In other words, dexterity and finger coupling may explain why we see the most slowing in the little finger and the least in the index finger.

Motor slowing in terms of the relative decrease in tapping speed was consistently present in the young and the aged participants and, interestingly, we observed no age differences. Indeed, we did find a marginally significant age difference (*p* = 0.048) when investigating the absolute decrease in tapping speed instead of the relative decrease. We propose however that this significance arose because of the different initial tapping speed between young and aged participants. Young tap faster than aged and hence they have the larger ‘capacity’ to decrease. We thus suggest that the relative decrease is a more reliable measure. This is also supported by the fact that the linear mixed effects model analysing the time course of motor slowing had a random intercept of participant (i.e., accounting for individual participant differences) and showed no significant *Age Group x Time* interaction.

In other fatiguing paradigms, dynamic tasks with unconstrained angular velocities reveal more fatigue in aged compared to young participants (Sundberg et al., 2019). It has been argued that this age difference may have been caused by greater metabolite accumulation in the muscle for aged compared to young participants (Paris et al., 2022; Sundberg et al., 2019). This peripheral factor may hence contribute more to the age difference compared to central factors. That peripheral factors may drive age differences more than central factors, is also supported by the findings that voluntary activation, which is an index for central fatigue, was similar between young and aged participants (Dalton et al., 2010, 2012, 2015; Sundberg et al., 2018). Thus, no significant age difference in our study may be because we assessed motor fatigability with a paradigm that relies more on supraspinal rather than peripheral mechanisms (Arias et al., 2015; Bächinger et al., 2019; Madinabeitia-Mancebo et al., 2020, 2021; Madrid et al., 2016, 2018; Rodrigues et al., 2009).

Nevertheless, our finding of no significant age differences in the decrease in tapping speed between young and aged participants are inconsistent with previous studies which investigated the influence of age on the decrease in movement speed during finger tapping. Previous studies reported a greater decrease in tapping speed over 30 s for young compared to aged participants (Hubel et al., 2013a) or no decrease in tapping speed for aged participants at all (Arias et al., 2012). Hubel et al. (2013a) argue that the aged participants may have started off tapping closer to their optimal speed as they tested motor slowing only during one trial lasting 30 s which was preceded by one brief familiarization phase of 10 s. Our study differed in that regard, as we emphasized that our participants tap as fast as possible, they performed multiple trials during the experiment, and they had *ad libitum* familiarization attempts. Similarly, Arias et al. (2012) attribute the absence of fatigue effects in their group of healthy elderly to a significantly lower overall tapping speed compared to their healthy young. Even though the average tapping speed is not explicitly reported in the studies of Hubel et al. (2013a) and Arias et al. (2012), visual inspection of their graphs revealed that our aged participants indeed tapped faster on average than their aged groups (approx. 4.5 Hz (Hubel et al., 2013a) and approx. 4.8 Hz (Arias et al., 2012) vs. 5.2 Hz (average tapping speed for the aged group in our study)). We found a decrease in tapping speed in our aged participants, despite a difference in tapping speed between young and aged for the index finger, which is also the finger that was investigated by Arias et al. (2012) and Hubel et al. (2013a). We attribute this difference to the possibility for an extended familiarization and clear instructions, which motivated even the aged participants to perform the task with maximum speed from the beginning of the tapping phase until the end.

Some neurological and especially neurodegenerative disorders may have their onset only in later stages in life. With an average age of 57.6 years in our older age group, we explored a rather ‘young’ older age group in our study. It is possible that more pronounced motor slowing (indicating high fatigability) can only be detected at older age, and it would be interesting to extend our approach to cohorts of 70+ years. However, unlike maximal tapping speed, relative motor slowing was remarkably robust across our two age groups which differed by at least 20 years. This suggests that the motor slowing paradigm with the relative decrease in tapping speed as the main measure might be suitable to detect abnormal levels of supraspinal motor fatigability, e.g., due to neurological and neurodegenerative disorders, independent of age-related effects on motor control.

In summary, we show that a relative decrease in tapping speed does not differ between age groups, indicating that supraspinal mechanisms involved in motor slowing of aged healthy individuals may be comparable to young. We further provide an interesting characterization in regard to differences in motor fatigability and maximal tapping speed between fingers. This study is therefore a starting point for providing appropriate comparisons to detect or monitor pathological motor fatigability.

## Data Availability

The original contributions presented in the study are publicly available. This data can be found here: https://doi.org/10.3929/ethz-b-000692991.
