# Supplementary material for: Finger-specific effects of age on tapping speed and motor fatigability
Source: Front Hum Neurosci. 2024 Sep 25;18:1427336. doi: 10.3389/fnhum.2024.1427336 (PMC11461208; doi:10.3389/fnhum.2024.1427336)
Supplement: Supplementary file 1 [file Data_Sheet_1.docx]

# Supplementary Material

## Supplementary Material 1


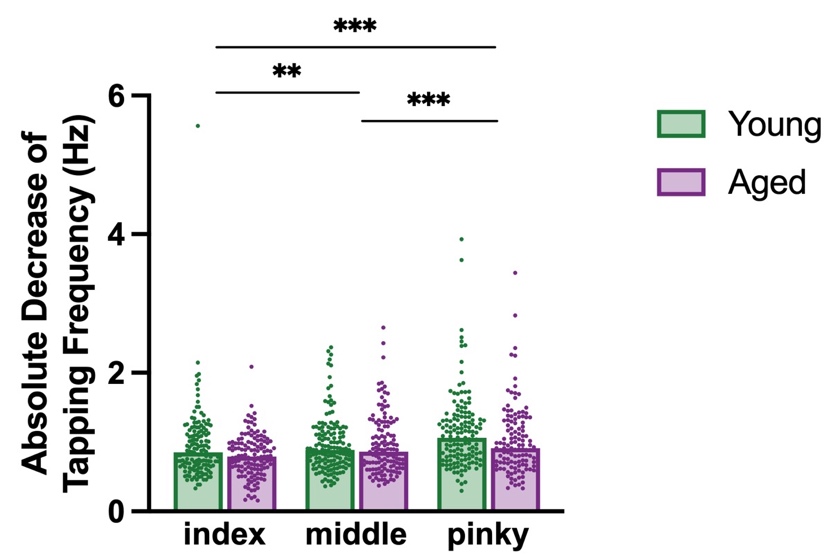


Supplementary Figure 1: Absolute decrease in tapping speed. There was a significant difference between Age Groups (F_(1, 327.03)_ = 3.9243, p < 0.05, $\eta_{p}^{2}$ = 0.01) with young decreasing more than aged, and a significant difference between fingers (F_(2, 488.99)_ = 32.216, p < 0.001, $\eta_{p}^{2}$ = 0.12) with significant post-hoc comparisons between finger pairs (post-hoc z $\geq$ 3.433), indicated by asterisk: ** p < 0.01, *** p < 0.001.

## Supplementary Material 2


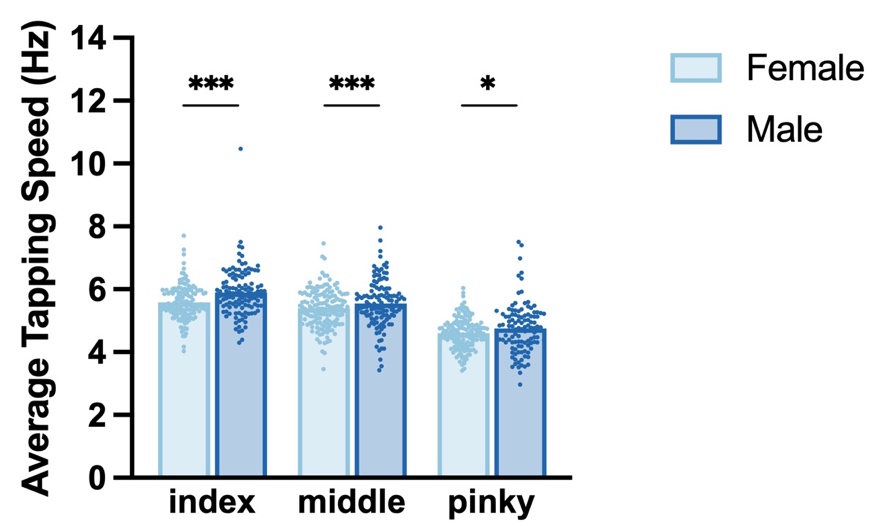


Supplementary Figure 2: Average tapping speed for female and male participants, split into fingers. Pairwise post-hoc comparisons revealed significant differences between female and male for all fingers (post-hoc |z| $\geq$ 2.101, p < 0.05) with males tapping faster than female.
